# Electrical impedance tomography for PEEP titration in ARDS patients: a systematic review and meta-analysis

**DOI:** 10.1007/s10877-025-01266-2

**Published:** 2025-02-26

**Authors:** Carlos Sanchez-Piedra, Begoña Rodríguez-Ortiz-de-Salazar, Oriol Roca, Francisco-Javier Prado-Galbarro, Lilisbeth Perestelo-Perez, Luis-Maria Sanchez-Gomez

**Affiliations:** 1 Health Technology Assessment Agency, Instituto de Salud Carlos III, Madrid, España; 2 RICAPPS. Red de Investigación en Cronicidad, Atención Primaria y Prevención y Promoción de la Salud, Madrid, Spain; 3 Servei de Medicina Intensiva, Parc Taulí Hospital Universitari, Institut d’Investigació i Innovació Parc Taulí (I3PT-CERCA), Sabadell, Spain; 4 Departament de Medicina, Universitat Autònoma de Barcelona, Bellaterra, Spain; 5 Ciber Enfermedades Respiratorias (Ciberes), Instituto de Salud Carlos III, Madrid, Spain; 6 Departamento de Investigación, Hospital Infantil de México Federico Gómez, Ciudad de México, México; 7 Evaluation and Planning Unit of the Canary Islands Health Service (SESCS), Tenerife, Spain; 8 IIS-IP. Instituto de Investigación Sanitaria. HU La Princesa, Madrid, Spain

**Keywords:** Electrical impedance tomography, Critical care, Mechanical ventilation, Acute respiratory distress syndrome, positive end-expiratory pressure

## Abstract

To assess the efficacy of electrical impedance tomography (EIT)-guided positive end-expiratory pressure (PEEP) titration in improving outcomes for patients with acute respiratory distress syndrome (ARDS). A systematic review and meta-analysis was conducted following Preferred Reporting Items for Systematic Reviews and Meta-Analyses (PRISMA) guidelines. Randomized controlled trials and observational studies with a control group comparing EIT-guided PEEP titration to other strategies were included. Endpoints analysed included mortality, days of mechanical ventilation (MV), intensive care unit (ICU) length of stay (LOS), weaning success rate, barotrauma, driving pressure (∆P), mechanical power (MP), Sequential Organ Failure Assessment (SOFA) score and adverse events. Pooled results were presented as Risk Ratio (RR) for dichotomous outcomes and standardized difference in means (SMD) for continuous outcomes. A total of 4 studies were identified (3 randomized controlled trials and one observational study). All studies were single-center studies (N total = 271 patients). The main limitations were related to potential bias in selecting reported outcomes. EIT-guided PEEP titration was associated with a significant reduction in mortality among critically ill patients with ARDS (RR = 0.64, 95% CI: 0.45–0.91). No significant differences were found in other outcomes. Our findings suggest that EIT may be a valuable tool for PEEP titration in critically ill patients with ARDS. By optimizing lung mechanics, EIT-guided PEEP titration may potentially reduce mortality rates. While larger, multicenter studies are needed to definitively establish the clinical role of EIT in ARDS management, our results provide promising evidence for its potential clinical impact.

**Supplementary Information:**

The online version contains supplementary material available at 10.1007/s10877-025-01266-2.

## Introduction

Acute respiratory distress syndrome (ARDS) is a severe inflammatory lung disease, which is prevalent among critically ill patients [1]. It is characterized by widespread inflammation and pulmonary edema that severely compromises respiratory function and it has a significant morbidity [2] and mortality [1]. Mechanical ventilation (MV) remains a life-support intervention for these patients. However, MV may have significant complications. It can lead to ventilator-induced lung injury (VILI) [3], a severe complication that exacerbates the preexisting lung injury and significantly contributes to increased mortality in ARDS patients. Therefore, optimizing ventilator settings to provide a more protective MV is essential.

In this clinical context, positive end-expiratory pressure (PEEP) is an essential element of MV. Optimizing PEEP is crucial for lung protection and reducing the risk of VILI while maintaining adequate oxygenation and ventilation. PEEP helps to prevent alveolar collapse and atelectrauma by maintaining alveolar distension and reducing cyclic opening and closing of lung units [4, 5]. Additionally, while PEEP can improve lung recruitment, leading to a more homogeneous distribution of ventilation and reduced stress on the alveolar wall [3, 6], it is important to note that the response of poorly aerated lung tissue to PEEP varies based on individual lung characteristics. In some cases, PEEP may increase the proportion of poorly aerated tissue, contributing to greater lung inhomogeneities and potentially exacerbating stress on the alveolar wall [7]. Therefore, individualizing PEEP strategies based on patient-specific factors, such as lung recruitability and dynamic compliance, is crucial to optimize its benefits and minimize potential harm.

The response to PEEP is highly heterogeneous among patients. Thus, assessing the individual PEEP response of each patient is warranted. PEEP aims to reduce the strain on the lung, which may be achieved in patients with higher lung recruitability, defined as the proportion of lung tissue in which aeration is restored at increased airway pressures. In contrast, when high PEEP is applied in non-recruitable patients, it increases the strain and the overdistension, promoting the occurrence of VILI. Different strategies have been used to set PEEP at the bedside [8]. One of these methods is electrical impedance tomography (EIT), which has been proposed as a potentially safe and effective alternative for bedside PEEP titration, although robust evidence supporting its safety and efficacy remains limited. EIT allows for non-invasive, radiation-free, bedside monitoring of regional ventilation [3, 9, 10]. It is based on the physical principle of impedance or the body’s ability to conduct an electric current [11, 12].

Despite growing interest in incorporating EIT into intensive care units (ICUs), the efficacy and safety of this technology in PEEP titration in mechanically ventilated patients with ARDS remains unclear. This systematic review aimed to assess the usefulness of PEEP titration guided by EIT on outcomes of critically ill patients with ARDS in comparison to other PEEP titration methods.

## Methods

### Data sources and search strategy

Three databases (MEDLINE through Ovid, EMBASE and the Cochrane Library) were searched from inception to February 1, 2024 (see electronic supplementary material 1, S.1). We searched for studies evaluating any other PEEP titration strategies and EIT-based individual PEEP setting of critical ARDS patients, with MeSH headings (“respiratory distress syndrome”, “critical care”, “artificial respiration”) and text words (ards, mechanical ventilation, intensive care, electrical impedance tomograph*). For the advanced search on MEDLINE and EMBASE, synonyms of the word “critical care” (intensive care) were combined with synonyms of the word artificial respiration (mechanical ventilation, assisted ventilation). The acronym “ARDS” was also considered. We searched for any additional studies in the references of all identified publications, including previous relevant meta-analyses and narrative reviews. Clinicaltrials.gov and PROSPERO databases were reviewed to find ongoing studies.

This systematic review was conducted in accordance with the Preferred Reporting Items for Systematic Reviews and Meta-Analyses (PRISMA) guidelines and registered on an international database of prospectively registered systematic reviews (PROSPERO) (CRD42024548293). The results were merged using the reference management software Ryaan.ai.

## Study selection

To define the systematic review, we used the acronym PICO (Participants, Interventions, Comparators, and Outcomes). The inclusion and exclusion criteria for study selection are presented in Table 1. For inclusion, any studies except reviews, case reports and conference abstracts or observational studies without a control group were included. We included randomized controlled trials (RCTs) and non-randomized studies (NRS) involving adult patients with ARDS on invasive mechanical ventilation. Studies in English or Spanish language were considered.

**Table 1:** Inclusion and exclusion criteria

Criteria	Inclusion	Exclusion
Participants	Adult patients with ARDS	Neonates, pediatrics, pregnant women, non-ARDS patients
Interventions	EIT-guided PEEP titration	Other PEEP titration strategies without EIT
Comparators	Any other PEEP titration strategies	N/A
Outcomes	Mortality, ICU LOS, ventilator-free days, SOFA score, driving pressure (∆P), mechanical power (MP), adverse events	Reviews, case reports, conference abstracts, studies without a control group
Study Design	Randomized controlled trials (RCTs), observational studies with a control group	Case-control studies, cohort studies without a control group
Language	English, Spanish	Other languages

N/A: Not applicable

Outcome variables related to the safety and efficacy of the intervention were assessed. Efficacy outcomes included were: mortality, ICU, hospital, days of admission to ICU, days with MV or ventilator-free days in the first 28 days of randomization, incidence of barotrauma/pneumothorax, SOFA (Sequential Organ Failure Assessment), variation in PEEP level, changes in ∆P and MP. The reporting of any adverse events (AE) during the study was assessed concerning to the safety of the interventions.

A clinical expert was consulted during the initial stages of protocol development to ensure the clinical relevance and feasibility of the research question and inclusion criteria. This involvement aligns with established methodological guidance for health technology assessment [13]. However, the expert did not participate in the selection of studies, data extraction, statistical analysis, or interpretation of results. This separation ensured that the findings remained unbiased and methodologically robust The screening and selection of studies were conducted independently by two review authors (CS-P and BR-O). These authors were blinded to the clinical expert’s input. Any disagreements between the two review authors were resolved by a third review author (LS-G), who was also blinded to the clinical expert’s input.

## Data extraction and assessment of bias

The data extraction process for the selected studies was conducted on an independent, peer-reviewed (CS-P and BR-O) basis. Any possible disagreements were resolved by consensus or in collaboration with another research team member (LS-G). A standardized data extraction form was developed to collect relevant information from the included studies. A dedicated Microsoft Excel^®^ spreadsheet was used to data extraction. The extracted data included data on study characteristics (e.g., study design, sample size, population characteristics), intervention details (e.g., EIT application method, PEEP titration strategy), outcome data, conclusions, and conflicts of interest.

Two authors (CS-P and BR-O) assessed study quality using the Cochrane Risk of Bias (RoB-II) assessment tool for RCTs and the Risk of Bias In Non-randomized Studies-of Interventions (ROBINS-I) for observational studies [14, 15]. Risk of bias was classified as low, of some concern, or high. Disagreements were resolved through consensus or with the participation of a third author (LS-G).

## Synthesis of results

Dichotomous outcomes (all-cause mortality and weaning success) were expressed as pooled Risk Ratio (RR) with 95% confidence intervals (CIs) and presented as a forest plot. For continuous outcomes (∆P), we reported the standardized difference in means (SMD). Statistical heterogeneity was assessed using the Chi-square test, where a p-value < 0.05 was interpreted as significant and evidence of heterogeneity. To assess the impact of statistical heterogeneity on the intervention effect, we calculated the I^2^ statistic. We used the Mantel–Haenszel random effects model to account for this heterogeneity. The following interpretation of I^2^ values was adopted: 0–40% (might not be important), 40–60% (moderate), 60–90% (substantial), and 75–100% (considerable). In addition to the primary analysis, a sensitivity analysis was conducted, restricting the included studies to RCTs to assess whether the observed effects were consistent when excluding NRS. It was not possible to assess potential publication bias because a minimum of ten studies were not selected.

The statistical analyses were performed by RevMan 5.0 software, The Nordic Cochrane Centre, Copenhagen, Denmark.

## Results

### Study identification

**Fig. 1:**
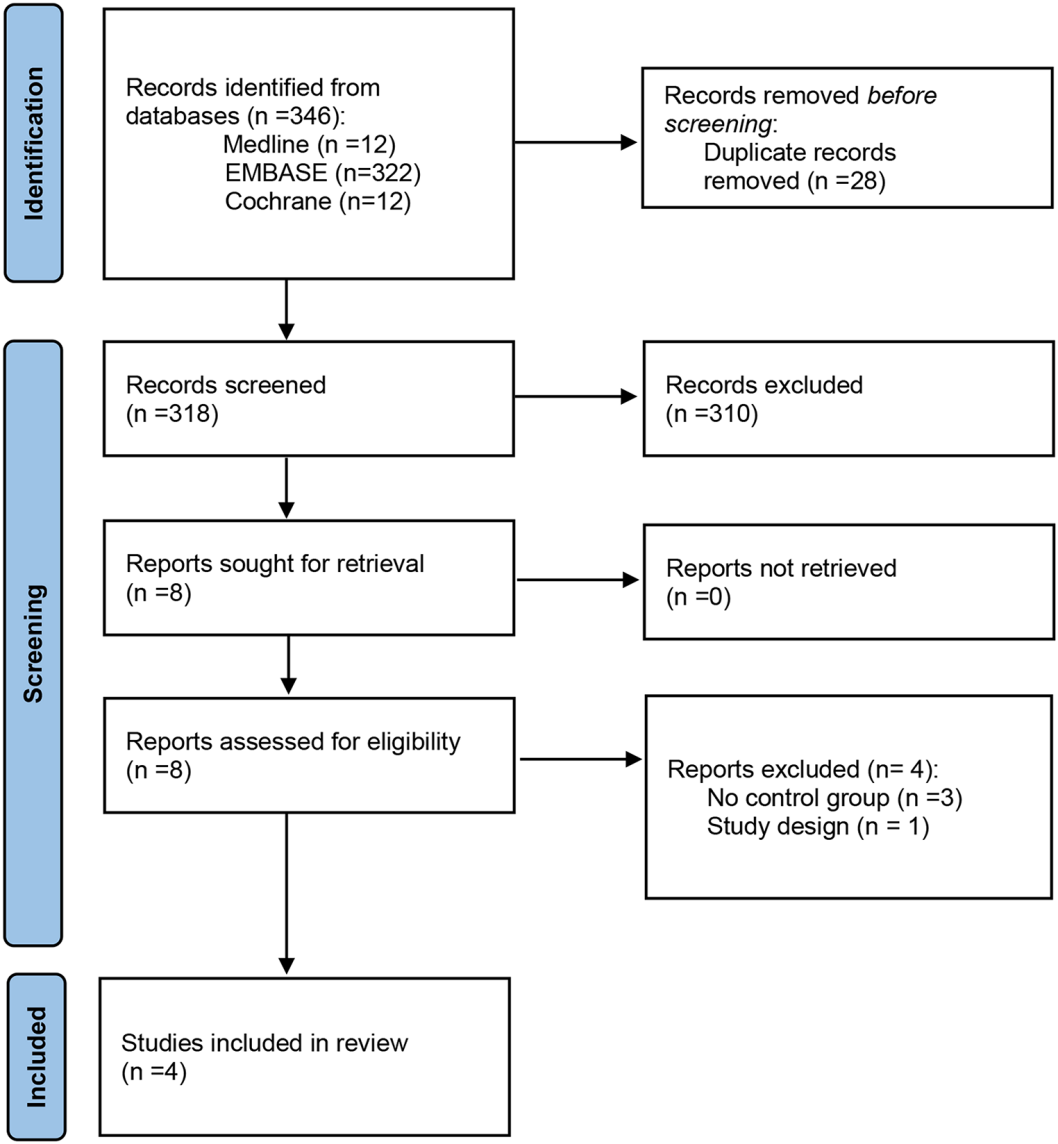
Study identification. PRISMA flow diagram

The search identified 346 records (MEDLINE = 12, EMBASE = 322, Cochrane Library = 12). A total of 318 unique citations were screened after eliminating duplicates. Of these, we excluded 310 records that did not meet selection criteria. Therefore, a total of 8 studies were selected for full-text review. A total of four studies were included in the systematic review and the meta-analysis (Fig. 1) [16–19]. And, 4 other studies were excluded. The main reasons for exclusion were study design (case series) [20], study objective (comparison of PEEP patterns [21] or selection of baseline PEEP [22, 23]) or no comparison group [20–23]. Electronic supplementary material 2, S.2 includes detailed information on the reasons for exclusion for each of these studies.

Our analysis detected two studies registered on ClinicalTrials.Gov (NCT05307913 and NCT05822869) that met the inclusion criteria for our PICO question and which were ongoing (no results available) at the time of this analysis [24].

### Description of the included studies and quality assessment

The included studies had different designs: 3 of the studies were RCTs (one of them with a crossover design) [16, 17, 19] and one study had an observational design [18]. All publications were in English. The total sample size of the included studies was 271 patients, with 133 involved in the EIT group compared to 138 in the control group. All studies used the same EIT device: Pulmovista 500 from Draeger Medical. All studies included patients with ARDS; in three of the studies, the diagnosis of ARDS was made according to the Berlin definition [25], and in the fourth study, it was made according to more specific clinical criteria [19]. In one of the studies, patients with moderate-severe ARDS were specifically selected. Characteristics and primary objectives of the four included studies are shown in Table 2.

**Table 2:** Characteristics and primary objective of the studies included in the meta-analysis

References	Year of publication	Design	Number of patients (intervention/control)	Period of realisation	Location	Control	Study objective	Funding
Zhao et al. [18]	2019	NRS	24/31	2016 (control group) 2017 (TIE)	Taiwan	PV curves	To examine the differences of EIT versus the routine procedure based on PV measurement obtained from the ventilator for PEEP setting.	Far Eastern Memorial Hospital (FEMH-2016-C-012 and FEMH-2017-C-055)
He et al. [16]	2021	RCT	61/56	Nov 2018 to Sep 2020	China	PEEP/FiO2 table	To determine whether individualised early titration of PEEP with EIT improved outcomes in patients with ARDS.	CAMS Innovation Fund for Medical Sciences (No. CAMS Innovation Fund for Medical Sciences (No. 2020-I2M-C&T-B-042), Capital’s Funds for Health Improvement and Research (No. 2020-2-40111), Excellence Program of Key Clinical Specialty of Beijing in 2020, and Beijing Municipal Science and Technology Commission (Grant No. Z201100005520051). National Natural Science Foundation of China (52077216).
Hsu et al. [17]	2021	RCT	42/45	April 2017 to Feb 2019	Taiwan	Point of maximal hysteresis in PV loop	To compare PEEP titration with EIT and ventilator-integrated PV in moderate to severe ARDS	Far Eastern Memorial Hospital (Grant Nos. FEMH-2018-C-077 and FEMH-2019-C-071), National Natural Science Foundation of China (Grant No. NSFC 51837011), Everest Program ofAFMU(Grant No. 2019ZFB002),BMBFMOVE(Grant No. FKZ 13FH628IX6) and H2020MCSA Rise (Grant No. 872488—DCPM).
Jiménez et al. [19]	2023	RCT	6/6	March 2019 to Jun 2022	United States	High PEEP/FiO2 table	To explore the effects of EIT-guided PEEP titration on mechanical power in patients with ARDS.	CHEST foundation.

**Fig. 2:**
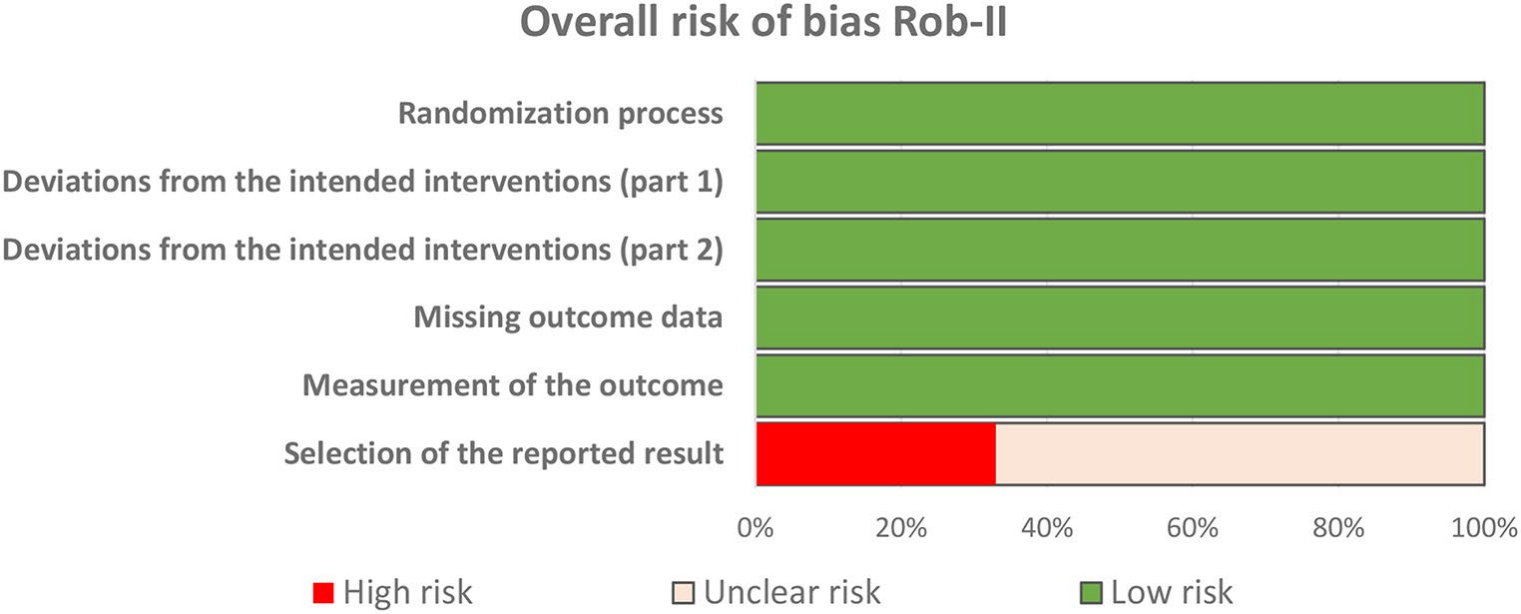
Risk of bias assessment for included randomized controlled trials (RCTs) using the Rob-II tool

Regarding quality assessment, two clinical trials were assessed as presenting an unclear risk of bias [12, 13], and another had a high risk of bias [14]. The main concerns in these studies were the risk of bias in the selection of the reported outcome (Fig. 2). In the case of the non-randomized study, a high risk of bias was identified, considering issues related to confounding factors and the selection of study participants [5].

## Mortality

**Fig. 3:**
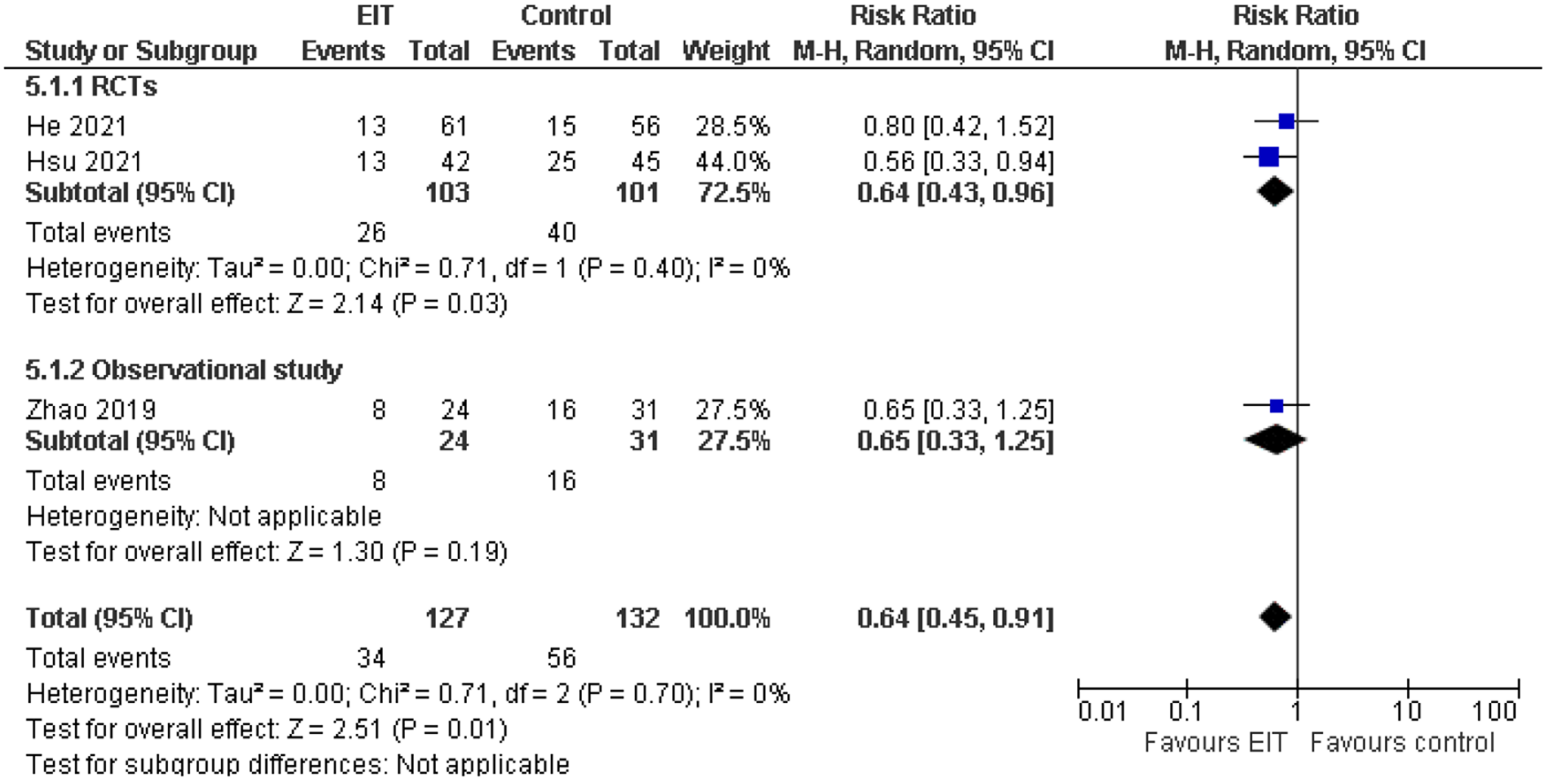
Comparison of mortality between EIT-guided and conventional PEEP titration. CI: Confidence Interval, df degrees of freedom, I^2^ heterogeneity statistic, M-H Mantel–Haenszel random effects model, RCTs: Randomized clinical trials

Three of the studies assessed all-cause mortality. The definition of the outcome variable was similar in all studies. While all studies showed higher mortality in the control group versus EIT group, only the RCT by Hsu et al. (RR = 0.56, 95% CI: 0.33–0.94) demonstrated a statistically significant difference that was clinically relevant. The pooled results for this variable are shown in Fig. 3. The overall random effects pooled bias was RR = 0.64, 95% CI: 0.45–0.91. Inter-study heterogeneity was low (I^2^ = 0%).

### Days on mechanical ventilation

Hsu et al. [17] assessed the number of days on MV and found no statistically significant difference between the two groups (20.6 ± 11.7 days in the control group versus 25.3 ± 14.4 days in the EIT group). He et al. [16]assessed ventilator-free days during the first 28 days of study participation and found no statistically significant difference in this outcome variable between the two groups: 14.0 (0–23.0) days in the EIT group versus 18.5 (0–24.0) days in the control group.

### Lenght of stay (LOS) in the ICU

Hsu et al. [17] and He et al. [16] evaluated the LOS in the ICU. None of the studies found statistically significant differences between the groups. In the study of Hsu et al. [17], a similar duration was observed in both groups: 19.9 ± 9.6 days in the control group versus 20.2 ± 9.1 days in the EIT group. In the study by He et al. [16], statistically significant differences between the groups could also not be demonstrated: 13.0 (7.0–25.0) in the EIT group versus 10.0 (7.0-14.8) in the control group.

### Weaning success rate

**Fig. 4:**
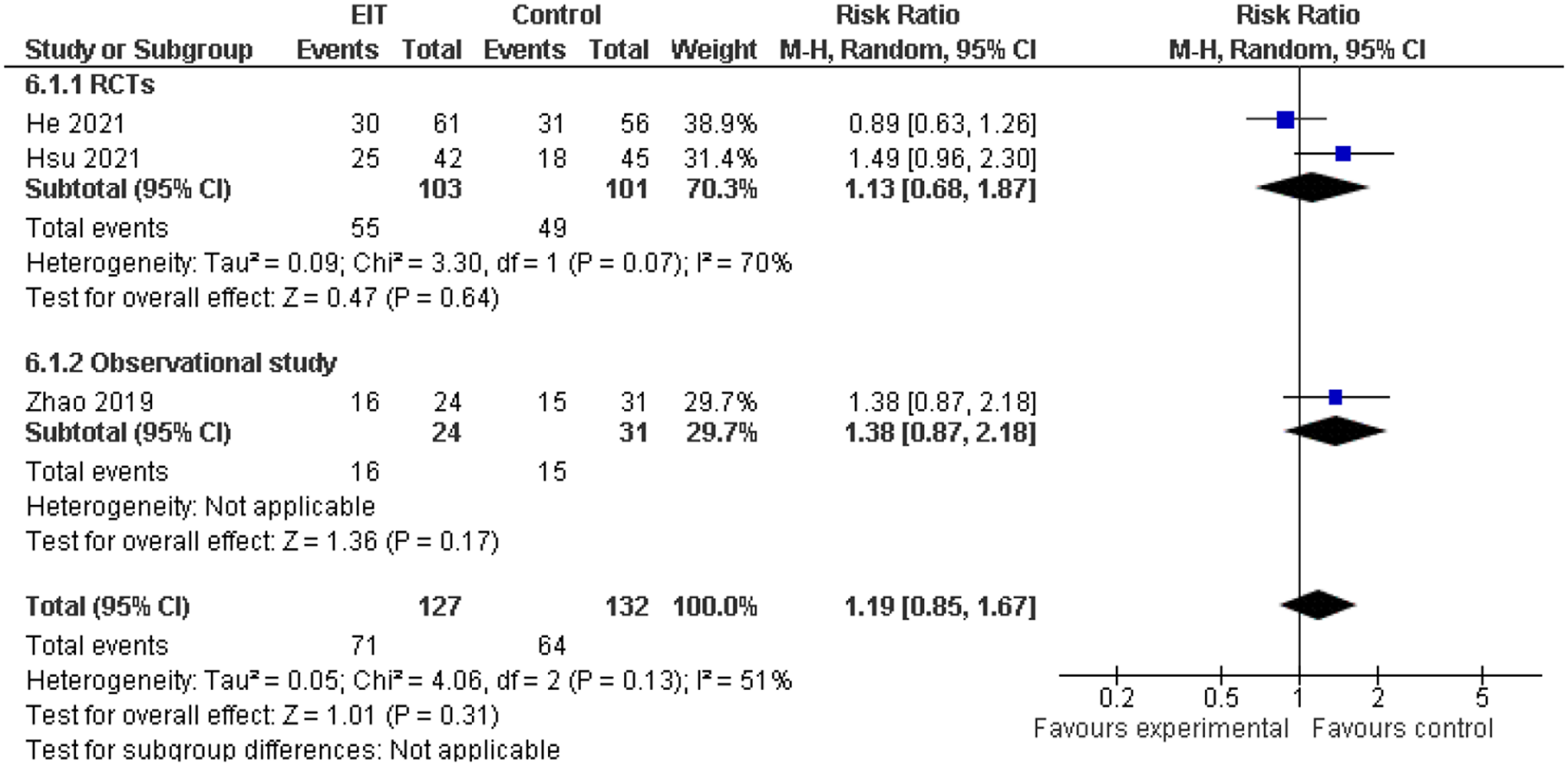
Comparison of successful weaning between EIT-guided and conventional PEEP titration. CI: Confidence Interval, df degrees of freedom, I^2^ heterogeneity statistic, M-H Mantel–Haenszel random effects model, RCTs: Randomized clinical trials

Three included studies assessed weaning success rate [16–18]. No significant differences were observed between the two groups in any of the individual studies. A meta-analysis of the available data revealed no statistically significant difference in weaning success rate between the EIT-guided PEEP titration and control groups: RR = 1.19, 95% CI: —0.85–1.67]. Inter-study heterogeneity was moderate (I^2^ = 51%) (Fig. 4).

### Occurrence of barotrauma/pneumothorax

The incidence of barotrauma was evaluated by two different studies [16, 18]. Only in one of them, two barotraumas were detected in the control group (2 in 31 patients, 6.5%) during the study [18]. No other group or study detected such events. Jimenez et al. [19] detected one pneumomediastinum, but it did not require any additional intervention.

### Driving pressure (∆P)

**Fig. 5:**
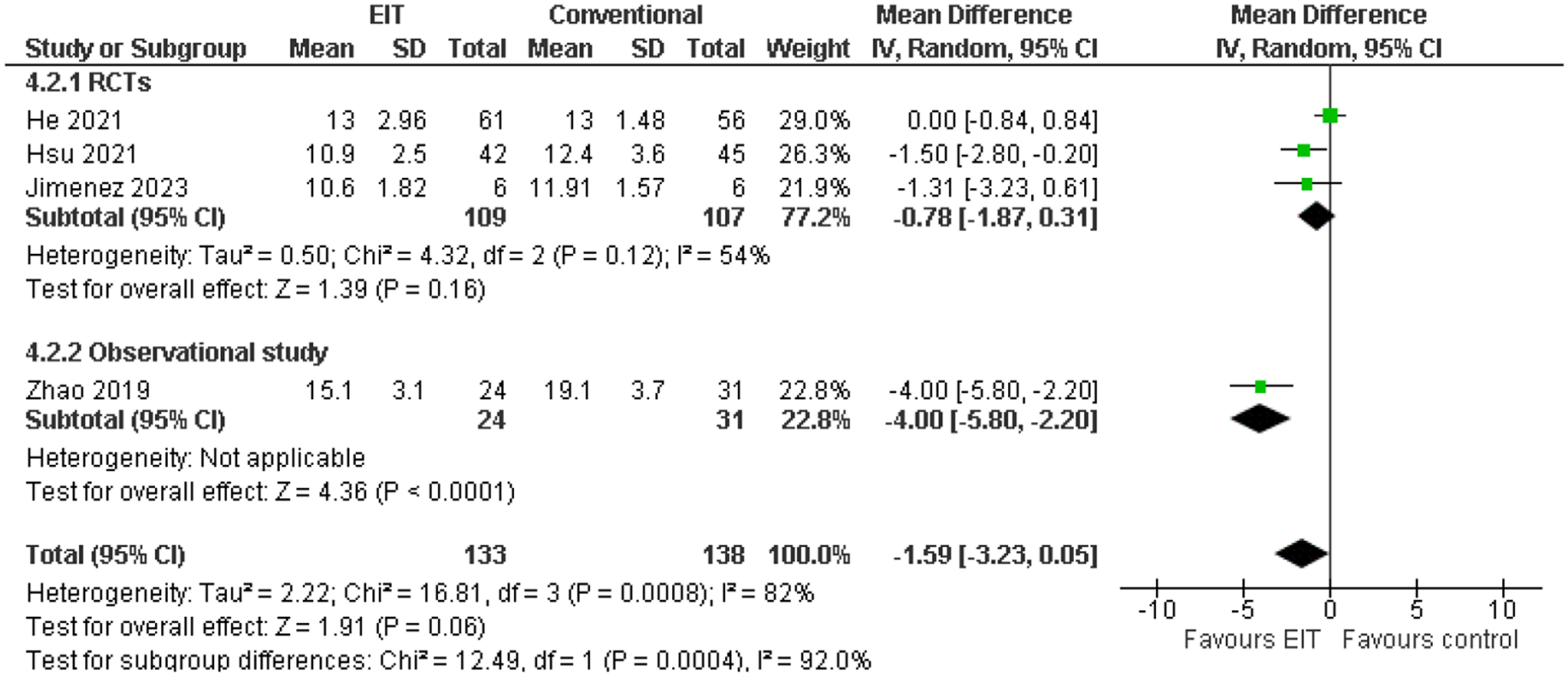
Comparison of ∆P between EIT-guided and conventional PEEP titration. CI: Confidence Interval, df degrees of freedom, I^2^ heterogeneity statistic, M-H Mantel–Haenszel random effects model, RCTs: Randomized clinical trials

All included studies assessed changes in driving pressure (∆P). While Zhao et al. [18] reported a significant reduction in ∆P in the EIT-guided PEEP group compared to the control group (15.1 ± 3.1 cmH2O vs. 19.1 ± 3.7 cmH2O), the remaining studies did not find statistically significant differences. Thus, the effect of EIT-guided PEEP on ∆P was not significantly different from comparators and heterogeneity (I^2^ = 82%) was detected in the pooled analysis (Fig. 5).

### Mechanical power (MP)

Only Jimenez et al. [19] measured the change in MP of an EIT-based strategy versus a strategy based on PEEP/FiO2 table. This study demonstrated a significant reduction in MP in the EIT group compared to the control group (− 2.50 ± 3.70 vs. 1.87 ± 1.61).

### SOFA (sequential organ failure assessment) score

SOFA score was measured only in the study by He et al. [16]. Differences in SOFA were measured after day 1 (difference in SOFA at day 1 minus SOFA measured at randomization) and after day 2 from randomization. Statistically significant differences were detected in the evolution of the SOFA index between baseline and day 3 in the EIT group versus control group.

### Safety

None of the selected studies has specifically assessed the safety of the intervention. There are no reports of discontinuation of the intervention due to AE in any of the included studies.

## Discussion

Our meta-analysis indicates that ARDS patients with optimized PEEP by EIT have a significantly lower mortality risk than those titrated with the conventional strategy. However, EIT was not associated with other clinical benefits. In this regard, significant heterogeneity was detected for ∆P and weaning success rate.

PEEP titration in ARDS patients with mechanical ventilation is a challenging task. Previous evidence has shown that individualized PEEP titration can improve overdistension and lung collapse resulting from prolonged use of mechanical ventilation, and ultimately reduce the incidence of VILI [20]. The pandemic has increased the number of studies conducted specifically in the COVID-19 population [20–22]. EIT has been proposed as a valuable technology for monitoring and guiding appropriate PEEP titration in critically ill patients with ARDS. Two previous meta-analyses analyzed the use of EIT-guided PEEP setting in ARDS patients and reported similar results [26, 27]. However, the present study presents substantial differences. Our analysis was more focused in terms of the design and purpose of the included studies. We excluded (1) studies due to differences in the study population [28, 29], (2) studies that assessed different outcomes not included in our PICO questions [21–23, 30–36], and (3) studies with inappropriate design, including those without a comparator group [20, 21, 23, 33, 37] (see electronic supplementary material 3, S.3). This strict design leads to an increase in the homogeneity of the observed results. This review provides additional insights not covered by Songsangvorn et al., including an economic perspective [38] and information on ongoing trials that may shape future research in this area. By addressing a more specific research question and providing additional data, this review complements existing literature and offers refined insights to guide clinical and research practices.

Notably, while He et al. excluded patients with COVID-19 and morbid obesity (Body Mass Index (BMI) > 40 kg/m2), these groups represent a significant proportion of ARDS patients with the highest risk of complications [37, 39]. Addressing this gap highlights the need for further research to evaluate PEEP titration strategies in these high-risk populations.

Previous studies arised the importance of mortality as a primary outcome to assess individualized PEEP titration in ARDS patients with MV [40, 41]. Our results showed a significant improvement in the EIT guided group versus conventional strategies, without heterogeneity. We have also evaluated ∆P and MP, outcomes that have been associated with mortality in ARDS patients [42].

The studies included in the meta-analysis did not report any relevant information on aspects related to the cost of EIT implementation. A previous review by the National Institute for Health and Care Excellence (NICE) briefly assessed the economics of this technology in 2019 [38]. Complete post-pandemic economic evaluation studies are needed to approximate better the economics associated with implementing this technology.

### Strengths and limitations

Our study presents some strengths. The research question targets a specific population, as well as intervention, comparators, and outcome variables. The aim was to evaluate the efficacy of the EIT guidance strategy versus conventional alternatives for titrating PEEP in critically ill patients with ARDS. We excluded any studies that were not focused on answering this research question. This analysis assessed mortality and weaning success as primary outcomes. Variables related to the performance of MV on lung mechanics, such as ∆P, were also evaluated. Detailed information on potential biases and the quality of the studies involved in the meta-analysis has also been included, and well-known and widely used tools have been used for the quality assessment.

However, it is not exempt from limitations. First, some degree of heterogeneity was reported in the pooled results of the weaning success and ∆P. This could be partially explained by differences in the baseline characteristics of the patients included in the selected studies. Differences in the conventional strategies used in the control groups and in the EIT-guided PEEP titration techniques may also have contributed to the inter-study variability. It is important to note that the I² statistic, while commonly used to assess heterogeneity, can be biased in small meta-analyses, potentially underestimating or overestimating true heterogeneity. This limitation should be considered when interpreting our findings [43]. Despite this, the I² statistic remains a widely accepted tool for assessing heterogeneity, and its use here aligns with standard practices in systematic reviews and meta-analyses. Secondly, the analysis was constrained by the small sample size, which limits the generability of the observed results. Only three single-center RCTs and one observational study were included, ranging from 12 to 117 patients, which may affect the precision and stability of the results. Finally, we found heterogeneity regarding the lack of standardization of PEEP assessment based on EIT among studies.

## Conclusion

Our results suggest that ARDS patients may benefit from EIT-guided PEEP titration. The real-time bedside assessment of regional ventilation provided by EIT may result in improved PEEP individualization, thereby limiting the occurrence of VILI and enhancing survival. However, standardization of EIT-guided PEEP selection tested in large RCTs is still needed. These trials should not only consider mortality, days free of MV, or length of stay but also economic and organizational issues that may be relevant to the implementation of the technology.

## Electronic supplementary material

Below is the link to the electronic supplementary material.

Supplementary Material 1

## Data Availability

No datasets were generated or analysed during the current study.
