# Supplementary material for: Electrical impedance tomography for PEEP titration in ARDS patients: a systematic review and meta-analysis
Source: J Clin Monit Comput. 2025 Feb 26;39(5):987–97. doi: 10.1007/s10877-025-01266-2 (PMC12474599; doi:10.1007/s10877-025-01266-2)
Supplement: Supplementary file 1 — Supplementary Material 1 [file 10877_2025_1266_MOESM1_ESM.docx]

**Supplementary Material**

**Index of supplementary material**

S.1) Search strategies. PUBMED, EMBASE and Cochrane.

S.2) Studies excluded after full-text screening: Reasons for exclusion.

S.3) Reasons for exclusion of studies from the SR of Sangsvorn et al.

**Supplementary material 1. Search strategies. PUBMED, EMBASE and Cochrane**

| **PUBMED** **Ovid MEDLINE(R) ALL <1946 to Febrero 5, 2024>** | | |
| --- | --- | --- |
| Nº | Query | Results |
| #1 | intensive care.ti,ab,kw. | 198437 |
| #2 | critical care/ | 61628 |
| #3 | 1 or 2 | 232460 |
| #4 | ards.ti,ab,kw. | 20064 |
| #5 | respiratory distress syndrome.ti,ab,kw. | 36896 |
| #6 | 4 or 5 | 42339 |
| #7 | artificial ventilation.ti,ab,kw. | 3069 |
| #8 | mechanical ventilation.ti,ab,kw. | 60689 |
| #9 | artificial respiration/ | 57645 |
| #10 | 7 or 8 or 9 | 97994 |
| #11 | 3 and 6 | 7708 |
| #12 | 10 and 11 | 3080 |
| #13 | electrical impedance tomograph*.ti,ab,kw. | 2304 |
| #14 | 12 and 13 | 22 |
| #15 | limit 14 to yr="2010 -Current" | 20 |
| #16 | limit 15 to humans | 12 |

| **EMBASE** | | |
| --- | --- | --- |
| Nº | Query | Results |
| 1 | 'intensive care'/exp | 932.676 |
| 2 | respiratory distress syndrome'/exp | 108.383 |
| 3 | #1 AND #2 | 40.496 |
| 4 | artificial ventilation':ti,ab,kw | 4.918 |
| 5 | mechanical ventilation':ti,ab,kw | 100.350 |
| 6 | assisted ventilation'/exp | 253.963 |
| 7 | #4 OR #5 OR #6 | 323.903 |
| 8 | #3 AND #7 | 26.275 |
| 9 | electrical impedance tomograph*':ti,ab,kw | 2.952 |
| 10 | electrical AND impedance AND tomograph | 185 |
| 11 | #9 OR #10 | 3.049 |
| 12 | #8 AND #11 | 394 |
| 13 | #8 AND #11 AND [humans]/lim | 322 |

| **COCHRANE** | | |
| --- | --- | --- |
| **Nº** | **Query** | **Results** |
| 1 | intensive care:ti,ab,kw | 40.981 |
| 2 | MeSH descriptor: [Critical Care] explode all trees | 3.065 |
| 3 | #1 OR #2 | 41.502 |
| 4 | MeSH descriptor: [Respiratory Distress Syndrome] explode all trees | 3.564 |
| 5 | ards:ti,ab,kw | 2.739 |
| 6 | #4 OR #5 | 5.211 |
| 7 | #3 AND #6 | 1.489 |
| 8 | MeSH descriptor: [Respiration, Artificial] explode all trees | 9.352 |
| 9 | artificial ventilation:ti,ab,kw | 10.956 |
| 10 | mechanical ventilation:ti,ab,kw | 14367 |
| 11 | #8 OR #9 OR #10 | 23.637 |
| 12 | #7 AND #11 | 1015 |
| 13 | electrical impedance tomograph*:ti,ab,kw | 354 |
| 14 | #12 AND #13 | 12 |

**Supplementary table 2. Studies excluded after full-text screening: Reasons for exclusion.**

| **Author/ year** | **Reference** | **Reason for exclusion** |
| --- | --- | --- |
| Gibot 2021 | Gibot S, Conrad M, Courte G, Cravoisy A. Positive End-Expiratory Pressure Setting in COVID-19-Related Acute Respiratory Distress Syndrome: Comparison Between Electrical Impedance Tomography, PEEP/FiO2 Tables, and Transpulmonary Pressure. Front Med (Lausanne). 2021;22(8):720920. | Design: case series. No control group. Design pre-test, post-test. |
| Caetano 2023 | Caetano DS, Morais CC, Leite WS, Lins RAC, Medeiros KJ, Cornejo RA, de Andrade AD, Campos SL, Brandão DC. Electrical Impedance Tomographic Mapping of Hypoventilated Lung Areas in Intubated Patients With COVID-19. Respir Care. 2023 Jun;68(6):773-776. | Brief article. No control group. Objective: patterns are compared. |
| Jonkman 2023 | Jonkman AH, Alcala GC, Pavlovsky B, Roca O, Spadaro S, Scaramuzzo G, Chen L, Dianti J, Sousa MLA, Sklar MC, Piraino T, Ge H, Chen GQ, Zhou JX, Li J, Goligher EC, Costa E, Mancebo J, Mauri T, Amato M, Brochard LJ; Pleural Pressure Working Group (PLUG). Lung Recruitment Assessed by Electrical Impedance Tomography (RECRUIT): A Multicenter Study of COVID-19 acute respiratory distress syndrome. Am J Respir Crit Care Med. 2023; 1;208(1):25-38. | No control group. The objective is to measure the range of recruitability, and the effects of PEEP, in order to select the optimal PEEP. |
| Somhorst 2022 | Somhorst P, van der Zee P, Endeman H, Gommers D. PEEP-FiO2 table versus EIT to titrate PEEP in mechanically ventilated patients with COVID-19-related ARDS. Crit Care. 2022 Sep 12;26(1):272. | No control group. The objective is to select the initial PEEP titration. |

**Supplementary table 3. Reasons for exclusion of studies from the SR of Sangsvorn et al.**

| **Study** | **Year** | **Reason for exclusion** |
| --- | --- | --- |
| Eronia et al | 2017 | No control group was identified. This was a feasibility study aimed at testing t the feasibility of setting PEEP with EIT to prevent lung de-recruitment following a recruitment maneuver. |
| Heines et al | 2019 | Clinical implementation study of the EIT, describing the experience in a single center. |
| Scaramuzzo et al | 2020 | The objective was to compare the physiologic effects of PEEP guided by EIT versus transpulmonary pressure in patients with ADRS. |
| Gibot et al | 2021 | Case series design. No control group was identified. |
| Becher et al | 2021 | This study was excluded because the objective differs from the research question and there is no control group. |
| Di Pierro et al | 2022 | This analysis was focused on EIT titration performed in patients Covid -ARDS versus No Covid -ARDS undergoing V-V ECMO support |
| Somhorst et al | 2022 | The objective is to select the initial PEEP titration and is limited to covid-19 patients. |
| Liu et al | 2022 | The population and comparator group do not answer our research question. The objective was to compare the respiratory mechanics in mechanical ventilated ARDS patients with or without COPD. |
| Jonkman et al | 2023 | The objective of this study differs from our research question. No data can be extracted from the control group. |
